# Association of ERCC1 rs11615 Polymorphism with the Risk of Cervical Cancer Especially in Chinese Populations: A Meta-Analysis

**DOI:** 10.1155/2022/1790993

**Published:** 2022-10-07

**Authors:** Yufeng Zhang, Yue Teng, Yuanjie Zhu, Yi Wu, Yuting Wen, Xinjian Liu, Dake Li

**Affiliations:** ^1^Department of Pathogen Biology, Nanjing Medical University, Nanjing 211166, China; ^2^Key Laboratory of Antibody Technique of National Health Commission of China, Nanjing Medical University, Nanjing 211166, China; ^3^Department of Gynecology, Nanjing Maternity and Child Health Care Hospital, Women's Hospital of Nanjing Medical University, Nanjing 210004, China

## Abstract

Abnormalities of the ERCC1 gene can affect DNA repair pathways, thereby having a vital effect on genomic stability. A growing amount of case-control studies have focused on making an investigation of the association between ERCC1 rs11615 polymorphism and cervical cancer susceptibility. However, the controversial results have raised concerns. To draw a more accurate conclusion, six studies were elaborately selected from the electronic databases for this meta-analysis, with 753 cervical cancer cases and 851 healthy controls. We applied pooled ORs combined with 95% CIs to test the potential associations. Significant associations were revealed in Chinese populations (T vs C: OR = 1.557 and 95%CI = 1.234–1.966; TT vs CC: OR = 3.175 and 95%CI = 1.754–5.748; TT/CT vs CC: OR = 1.512 and 95%CI = 1.126–2,031; and TT vs CT/CC: OR = 2.836 and 95%CI = 1.592–5.051). Even when the studies deviating from HWE were excluded, an increased cervical cancer susceptibility was observed in Chinese. These results disclose that there is an obvious correlation between the risk of cervical cancer and ERCC1 rs11615 polymorphism, especially in Chinese populations, and the T variant is the risky one. Also, our findings need further studies to validate.

## 1. Introduction

Cervical cancer is the fourth most frequently diagnosed malignant tumor in clinical practice [1], and it is also a gynecological tumor with a fatality rate second only to breast cancer among women [2]. Unfortunately, the specific pathogenesis of cervical cancer has not been expounded at present. Existing studies have made clear that HPV infection is an important factor leading to cervical cancer [3]. Nevertheless, HPV infection alone is not enough to cause the occurrence of cervical cancer. It is also affected by host genetics, age, sexual life, chronic vaginitis, and other factors [3–5]. Among them, genes have a crucial impact on determining cancer risk, and multifarious genetic variants also increase the risk of suffering from cancer [6].

DNA damage refers to changes in DNA composition and structure caused by various internal and external factors, including ionizing radiation, ultraviolet radiation, and free radicals [7, 8]. Therefore, for the sake of maintaining the integrity and stability of the genome, organisms must develop a series of DNA repair pathways involving a variety of complex mechanisms [9, 10]. These pathways require the participation of multitudinous proteins, and without doubt, changing genes that encode these proteins will affect the expression level of related proteins, leading to direct damage to DNA repair ability, while the gene mutation and chromosome damage caused by incomplete DNA repair are important factors of cancer transformation and tumor progression [11–13].

As a DNA repair protein, excision repair cross-complementation group 1 (ERCC1) participates in numerous DNA damage repair pathways, such as nucleotide excision repair (NER), basic excision repair (BER), inter-strand cross-linking (ICL) repair, and recombinant DNA repair [14–17]. Genetic mutations occurring on a single nucleotide can cause DNA sequence polymorphism, including the insertion, deletion, transformation, and replacement of a single base, which is known as single nucleotide polymorphism (SNP). SNPs are reported to be the most usual form of genetic variation in humans, and the proportion is more than 90% [18, 19].

In the past, many literatures have studied the effect of ERCC1 polymorphism on cancers, especially ERCC1 rs3212986 and rs11615. For example, Bajpai et al. [20] have reported the relationship between ERCC1 rs3212986 polymorphism and cervical cancer susceptibility and found that the T variant at this site is correlated with the occurrence of cervical cancer. Nevertheless, more studies have focused on the association between ERCC1 rs11615 and cancer. The study by Yu et al. [21] has shown that ERCC1 C19007T (ASN118ASN, rs11615) polymorphism may lead to decline protein expression by affecting its mRNA and is ultimately associated with decreased DNA repair ability in cancer cells [22]. In addition, studies have shown that individuals with reduced ERCC1 expression may have a higher risk of the squamous intraepithelial lesion, which ultimately leads to invasive cervical cancer [23]. It is worth noting that low ERCC1 expression is also associated with poor prognosis in cervical cancer patients [24]. Therefore, we hypothesized that ERCC1 rs11615 polymorphism might play a role in cervical cancer susceptibility and prognosis.

Based on this hypothesis, we searched the literatures and found that the correlation between ERCC1 rs11615 polymorphism and susceptibility to cancers has been reported in multiple meta-analyses, such as breast cancer, lung cancer, pancreatic cancer, head-and-neck cancer, and colorectal cancer [14, 25–28]. However, case-control studies or meta-analysis about cervical cancer have yielded conflicting results [6, 29]. In consequence, based on existing case-control studies, we carried out this new meta-analysis, aiming to elucidate the correlation between ERCC1 rs11615 polymorphism and cervical cancer susceptibility and better predict the occurrence and development of cervical cancer in clinical practice.

## 2. Methods

### 2.1. Literature Retrieval Strategy

All relevant studies were independently searched and screened by two researchers. By entering the following keywords: ‘cervical cancer' or ‘cervical carcinoma' or ‘cervical neoplasm' and ‘SNP' or ‘polymorphism' or ‘genetic variant' and ‘ERCC1' or ‘excision repair cross-complementary group 1' or ‘rs11615', we retrieved correlative articles published in the CNKI, Embase, Pubmed, WanFang, and EBSCO databases up to September 2021. Additionally, we attempted to find potentially relevant studies from the abstracts, full texts, and reference lists of the identified articles.

### 2.2. Selection and Exclusion Criteria

Articles selected for our meta-analysis should accord with the following criteria: (1) evaluating the correlation between ERCC1 rs11615 polymorphism and cervical cancer susceptibility; (2) human case-control study; and (3) containing complete genotyping data for calculation. Articles with repeated published data or which were case reports or conference reports or reviews and where genotype frequency or allele frequency could not be obtained were excluded. After identifying the original studies required, we performed a quality control assessment of the literature by using Newcastle-Ottawa Scale (NOS).

### 2.3. Data Extraction

Two researchers extracted the original data according to the selection criteria, and a third researcher assisted them if the collected data were found to be inconsistent. The information we collected was as follows: (1) first author; (2) published year; (3) country and ethnicity; (4) case number and control number; (5) genotyping method; (6) allele and genotype frequency; and (7) Hardy-Weinberg equilibrium (HWE) in controls (it can be calculated from the control data).

### 2.4. Statistical Analysis

In this study, almost all the analysis processes were accomplished by the STATA 12.0 software. A Chi-square test for the genotypes frequencies of controls was applied to judge whether it is in keeping with HWE; *P* < 0.05 indicates that HWE is not balanced [30]. We used pooled odds ratios (ORs) combined with their 95% confidence intervals (CIs) to estimate the association in allele (T vs C), homozygous (TT vs CC), heterozygous (TC vs CC), recessive (TT vs TC/CC), and dominant (TC/TT vs CC) models. According to the characteristics of the literatures, we chose country and genotype methods as the targets of subgroup analysis. Moreover, we used the *I*^2^ test to detect heterogeneity, with *I*^2^ > 50% indicating significant heterogeneity, and Q-test with a *P* < 0.1 indicating that. When the result showed *P* > 0.1 and *I*^2^ < 50%, it means that no or low heterogeneity was found and a fixed-effects model could be utilized. If not, a random-effects model was selected [31, 32]. Furthermore, sensitivity analysis, which can detect the impact of each literature on the final results, and publication bias, which was usually shown by Begg's funnel plot and Egger's test, were also proceeded by Stata. If *P* < 0.05, it can be regarded as there is significant bias [33]. Finally, we conducted an additional trial sequential analysis by using TSA software to verify whether our existing sample size was sufficient to support our conclusions.

## 3. Results

### 3.1. Characteristics of Studies

**Figure 1:**
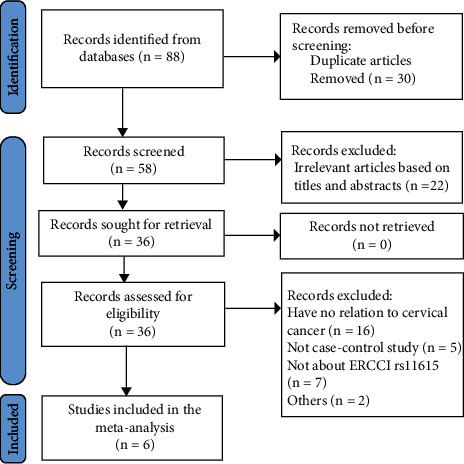
The detailed process of literature selection and inclusion.

All the related scripts and supported data were uploaded on the Github page https://github.com/ZYFNJMU/ERCC1-rs11615-and-cervical-cancer. The detailed procedure of literature selection and inclusion is shown in Figure 1. In the end, we retrieved six suitable original pieces of literature for this meta-analysis [22, 29, 34–37], involving 753 cervical cancer cases and 851 controls.  Table 1 showed the result of the quality control assessment conducted through NOS. It indicated that the quality of the six original studies included in our meta-analysis was relatively excellent. As for subjects, all of them were Asians. In Tables 2 and 3, we, respectively, exhibited the characteristics of all the contained studies and the frequency distribution of alleles and genotypes for each study.

### 3.2. Quantitative Data Synthesis

**Figure 2:**
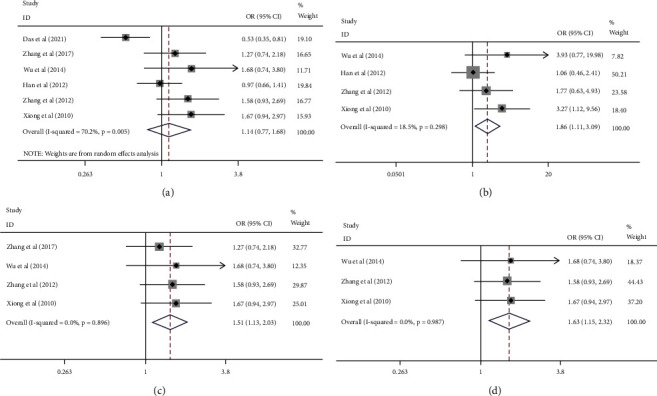
Forest plots of the association between cervical cancer and ERCC1 rs11615. (a) A random-effects model of six studies (TT/CT vs CC). (b) A fixed-effects model of four studies conformed to HWE (TT vs CT/CC). (c) A fixed-effects model of four studies about Chinese populations (TT/CT vs CC). (d) A fixed-effects model of three studies about Chinese populations conformed to HWE (TT/CT vs CC).

Table 4 summarized the main results of rs11615 polymorphism and cervical cancer susceptibility. The results announced that cervical cancer susceptibility is not associated with ERCC1 rs11615 polymorphism in all five models (Figure 2(a)). Meta-analysis was conducted again after excluding studies that do not conform to HWE, and the results showed that there is an explicit association between them (Figure 2(b)).

### 3.3. Subgroup Analysis

**Figure 3:**
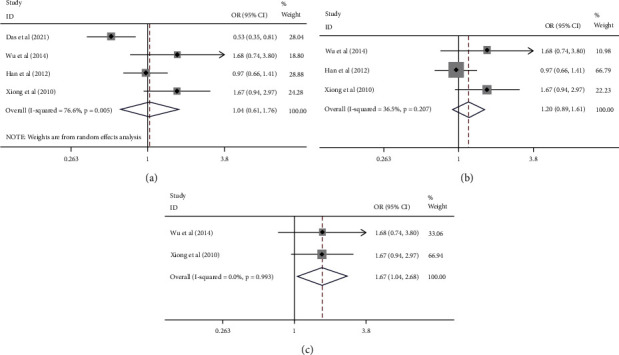
Forest plots of the association between cervical cancer and ERCC1 rs11615. (a) A random-effects model of four studies genotyped by PCR-RFLP (TT/CT vs CC). (b) A fixed-effects model of three studies conformed to HWE and was genotyped by PCR-RFLP (TT/CT vs CC). (c) A fixed-effects model of two studies about Chinese conformed to HWE and genotyped by PCR-RFLP (TT/CT vs CC).

In the subgroup analysis based on country classification, four case-control studies on Chinese people were included. Since there are only two studies, respectively, for the people of Bangladesh and Korea, no subgroup analysis of Bangladeshi and Korean populations was performed. The results showed a growing risk of cervical cancer in the Chinese population in four models but not in the heterozygous model (Table 4 and Figure 2(c)). We removed the study that did not conform to HWE once again, and the results still showed the prominent correlation for Chinese in the four models (Table 4 and Figure 2(d)). Four studies genotyped by PCR-RFLP were applied for subgroup analysis, and the results stated that there was no association between them, regardless of whether we removed the studies that did not conform to HWE or not. Among the four studies, there were two articles about Chinese. Therefore, we conducted an analysis again using the two articles. In the end, we also found that they are associated. (Figure 3).

### 3.4. Detection of Heterogeneity

The results of heterogeneity testing are summarized in Table 4. The fixed-effects model was applied if *P* > 0.1 and *I*^2^ < 50%; otherwise, we chose a random-effects model. When no studies were excluded, we found significant heterogeneity in six studies (heterogeneity test results were, respectively, equal to 80.2%, 72.3%, 44.7%, 70.2%, and 66.1%). In all analyses only about Chinese populations, we found there was nearly no heterogeneity.

### 3.5. Sensitivity Analysis and Publication Bias

**Figure 4:**
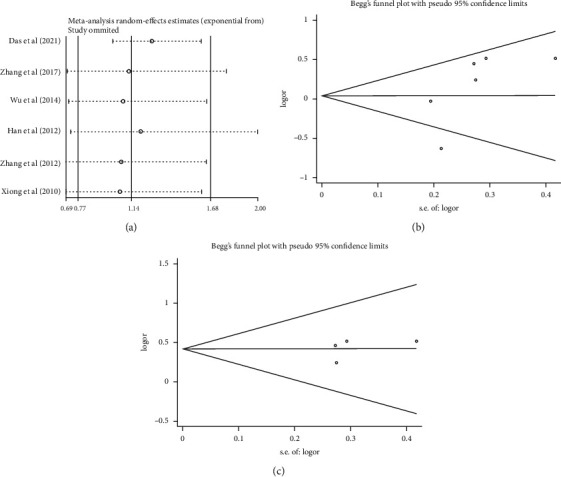
(a) Sensitivity analysis of six studies (TT/CT vs CC). (b, c) Funnel plots for publication bias. (b) All studies (TT/CT vs CC). (c) Studies of Chinese (excluded Das's and Han's) (TT/CT vs CC).

As mentioned earlier, sensitivity analysis was carried out by eliminating individual studies once to research the impact of individual studies on the whole. It can be seen that Das's study has a great influence on several models and Han's has a little influence (Figure 4(a)). The results of publication bias are shown in Table 4 and Figures 4(b) and 4(c). They indicated no publication bias in all situations.

### 3.6. Trial Sequential Analysis

**Figure 5:**
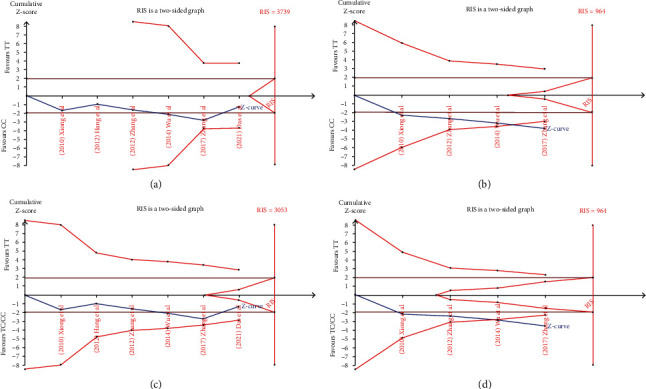
(a, b) Trial sequential analyses of TT vs CC model. (a) All six literatures were included. (b) Four literatures about Chinese were included. (c, d) Trial sequential analyses of TT vs CT/CC model. (c) All six literatures were included. (d) four literatures about Chinese were included.

As shown in Figure 5, we conducted trial sequential analyses under all gene models. When all six literatures were included, the *Z*-curve sometimes crossed the traditional boundary, but never crossed the TSA boundary and required information size (RIS), suggesting the possibility of false positive errors. However, when only four literatures about Chinese were included, the *Z*-curve passed the traditional boundary and TSA boundary, suggesting that the results of the meta-analysis were stable, even if they did not reach RIS.

## 4. Discussion

Abnormal mechanisms of cell proliferation, differentiation, and death can lead to tumors. The specific mechanisms of their occurrence and development have always been the focus of research. However, as is known to all, abnormal gene structure, DNA damage, and abnormal expression or function of tumor genes and tumor suppressor genes are important preconditions for malignant transformation [38]. Therefore, DNA repair acts as a practically protective response to maintain the stability of the cell genome. Various DNA repair mechanisms, such as NER, BER, mismatch repair, and recombination repair mechanisms, control DNA damage [39]. ERCC1 is a pivotal regulatory factor in the nucleotide excision repair (NERs) pathway [40], and genetic variations in this gene can affect DNA repair [41, 42].

SNPs are a common type of heritable variation in humans, and rs11615 (C > T) is a familiar polymorphism of the ERCC1 gene. As mentioned above, many meta-analyses have been reported about rs11615 and cancer. Most of the results show a significant correlation between multiple cancers and this mutation locus. For cervical cancer, Ma et al. [6] included data from case-control studies of Han et al. [34] and Xiong et al. [22], and the results showed there was no association, but Ma et al. did not focus on this issue.

This meta-analysis is aimed at making an investigation into the correlation between ERCC1 rs11615 polymorphism and the risk of cervical cancer. In the process of literature searching, we were surprised to find a Genome-Wide Association Studies (GWAS) about cervical cancer from the Swedish population, which also reported the relationship between ERCC1 rs11615 and cervical cancer susceptibility, but unfortunately, it was difficult to obtain allele and genotype data from GWAS of cases and controls [43]. Therefore, these studies were not included in our meta-analysis. At last, six eligible articles were retrieved for this study, involving 753 cases and 851 controls. But there turned out to be no association between them in all five gene models. During the process, we noticed that the control group data of two pieces of literature did not conform to HWE. Many possibilities can lead to deviations from HWE, such as genotyping errors and inbreeding, which may lead to our studies revealing erroneous conclusions. Therefore, we eliminated these data for further analysis, and ultimately in the allele model, homozygous model, and recessive model, we found significant correlations. But it is not reflected in the other two models, which may indicate that people with the TT genotype have a higher susceptibility to cervical cancer. In the subgroup analyses by country and genotype method, we revealed the more obvious association between them in Chinese populations. This association was significant even when we removed studies that deviate from HWE.

In addition, after sensitivity analysis, we found that Das's and Han's studies had a greater impact on the overall results, possibly because their case number was relatively larger compared to other studies, and they targeted, respectively, Bangladeshis and Korean people. They may also be the source of heterogeneity in our analysis. Our analysis after the exclusion of both Das's and Han's studies showed a stronger association and significantly reduced heterogeneity. This also reveals that our results are reliable for the Chinese. Unfortunately, other ethnic groups were not included in our analysis. As a result, the association of different ethnic groups is not fully understood at this time. Furthermore, we found no proof of publication bias in this study. Finally, our trial sequential analyses also suggested that the results of the meta-analysis were stable in Chinese populations.

**Figure 6:**
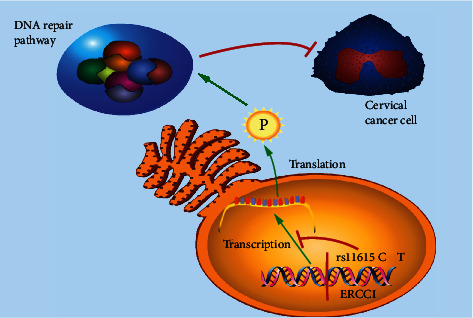
The mechanism between ERCC1 rs11615 polymorphism and cervical cancer.

Therefore, based on the results of our meta-analysis and existing research reports, we believe that ERCC1 rs11615 polymorphism may reduce DNA repair ability by affecting its mRNA stability and protein expression level, leading to an increased risk of cervical cancer (Figure 6). ERCC1 has the opportunity to be a target for cervical cancer diagnosis or drug therapy. Of course, the specific mechanism needs to be further explored.

Certainly, limitations still exist in this meta-analysis. First, only six pieces of literature were included, with a relatively low case number; therefore, the risk assessment of ERCC1 rs11615 polymorphism was not clear enough. In addition, most of the data of cases and controls provided are from Chinese populations, and there is no information such as age, environmental factors, and sex, which cause it difficult for us to conduct more subgroup analyses.

## 5. Conclusion

In brief, this meta-analysis put forward a conclusion that the ERCC1 rs11615 polymorphism increases cervical cancer susceptibility, especially in the Chinese populations, while TT genotype may have a higher risk. To promote in-depth research on genetic susceptibility to cervical cancer, more randomized controlled studies and systematic reviews with large samples, rigorous design, multicenter, and multilink will be of great importance and far-reaching significance.

## Figures and Tables

**Table 1:** The result of NOS.

Study	Selection				Comparability	Exposure			Scores
	Adequate definition of cases	Representativeness of cases	Selection of controls	Definition of controls	Consider comparability of cases and controls	Survey and evaluation methods of exposure	Using same method for ascertaining exposure of cases and controls	No response rates	
Das et al. [37]	★	★		★	★	★	★	★	**7**
Zhang et al. [36]	★	★	★	★	★	★	★	★	**8**
Wu et al. [35]	★	★	★	★	★★	★	★	★	**9**
Han et al. [34]	★	★	★	★	★	★	★	★	**8**
Zhang et al. [29]	★	★	★	★	★★	★	★	★	**9**
Xiong et al. [22]	★	★	★	★	★★	★	★	★	**9**

**Table 2:** Characteristics of the included studies.

Study	Year	SNP	Country	Ethnicity	Cases	Controls	Genotype method
Das et al.	2021	rs11615	Bangladesh	Asians	210	200	PCR-RFLP
Zhang et al.	2017	rs11615	China	Asians	95	121	MALDI-TOF
Wu et al.	2014	rs11615	China	Asians	48	48	PCR-RFLP
Han et al.	2012	rs11615	Korea	Asians	229	204	PCR-RFLP
Zhang et al.	2012	rs11615	China	Asians	80	175	PCR
Xiong et al.	2010	rs11615	China	Asians	91	103	PCR-RFLP

**Table 3:** The frequency distribution of alleles and genotypes.

Study	Cases			Controls			Cases		Controls		*P* for HWE
	CC	CT	TT	CC	CT	TT	C	T	C	T	
Das et al.	155	45	10	120	60	20	355	65	300	100	0.0047
Zhang et al.	43	42	10	62	55	4	128	62	179	63	0.0474
Wu et al.	25	16	7	31	15	2	66	30	77	19	0.9133
Han et al.	131	85	13	115	78	11	347	111	308	100	0.6349
Zhang et al.	39	34	7	105	61	9	112	48	271	79	0.9709
Xiong et al.	47	31	13	66	32	5	125	57	164	42	0.6627

**Table 4:** The main results of the meta-analysis.

Groups (quantity of studies)	Cases/controls	Genetic model	Effects model	Test of association			Test of heterogeneity		Begg's		Egger's	
				OR	95% CI	*P*	*I* ^2^ (%)	*P*	*z*	*P*	*t*	*P*
Overall (6)	753/851	T vs C	Random	1.199	0.819-1.754	0.351	80.2	0.000	1.88	0.060	1.77	0.152
		TT vs CC	Random	1.720	0.757-3.908	0.195	72.3	0.003	1.50	0.133	2.98	0.041
		TC vs CC	Fixed	0.992	0.800-1.229	0.938	44.7	0.108	0.75	0.452	1.32	0.256
		TT/CT vs CC	Random	1.140	0.774-1.680	0.506	70.2	0.005	1.13	0.260	1.83	0.142
		TT vs CT/CC	Random	1.633	0.789-3.379	0.187	66.1	0.012	1.50	0.133	3.00	0.040

China (4)	314/447	T vs C	Fixed	1.557	1.234-1.966	0.000	0.0	0.806	1.02	0.308	1.45	0.283
		TT vs CC	Fixed	3.175	1.754-5.748	0.000	0.0	0.840	1.02	0.308	1.17	0.361
		TC vs CC	Fixed	1.308	0.958-1.786	0.091	0.0	0.893	0.34	0.734	0.10	0.927
		TT/CT vs CC	Fixed	1.512	1.126-2.031	0.006	0.0	0.896	0.34	0.734	0.67	0.571
		TT vs CT/CC	Fixed	2.836	1.592-5.051	0.000	0.0	0.768	1.70	0.089	1.15	0.370

Conform to HWE (*P* > 0.05)												
Overall (4)	448/530	T vs C	Random	1.395	1.015-1.918	0.040	51.9	0.101	1.02	0.308	2.60	0.121
		TT vs CC	Fixed	1.983	1.176-3.344	0.010	30.8	0.227	1.02	0.308	2.04	0.178
		TC vs CC	Fixed	1.175	0.895-1.543	0.245	0.0	0.561	-0.34	1.000	1.45	0.284
		TT/CT vs CC	Fixed	1.280	0.989-1.657	0.061	23.4	0.271	0.34	0.734	2.24	0.155
		TT vs CT/CC	Fixed	1.855	1.113-3.093	0.018	18,5	0.298	1.02	0.308	2.07	0.174

China (3)	219/326	T vs C	Fixed	1.647	1.244-2.181	0.000	0.0	0.779	0.00	1.000	0.96	0.514
		TT vs CC	Fixed	3.004	1.543-6.008	0.001	0.0	0.682	1.04	0.296	0.83	0.558
		TC vs CC	Fixed	1.414	0.972-2.058	0.070	0.0	0.960	1.04	0.296	-1.17	0.450
		TT/CT vs CC	Fixed	1.630	1.146-2.319	0.007	0.0	0.987	0.00	1.000	0.80	0.570
		TT vs CT/CC	Fixed	2.661	1.374-5.154	0.004	0.0	0.616	1.04	0.296	0.84	0.555

## Data Availability

All data are available from the included literatures.
